# Supplementary figures and images for: Structures of NF-κB p52 homodimer-DNA complexes rationalize binding mechanisms and transcription activation
Source: eLife. 2023 Feb 13;12:e86258. doi: 10.7554/eLife.86258 (PMC9991059; doi:10.7554/eLife.86258)

His-Bcl3  
(1-446)

p52(1-327)

His-Bcl3  
(1-446)  
+  
p52(1-327)

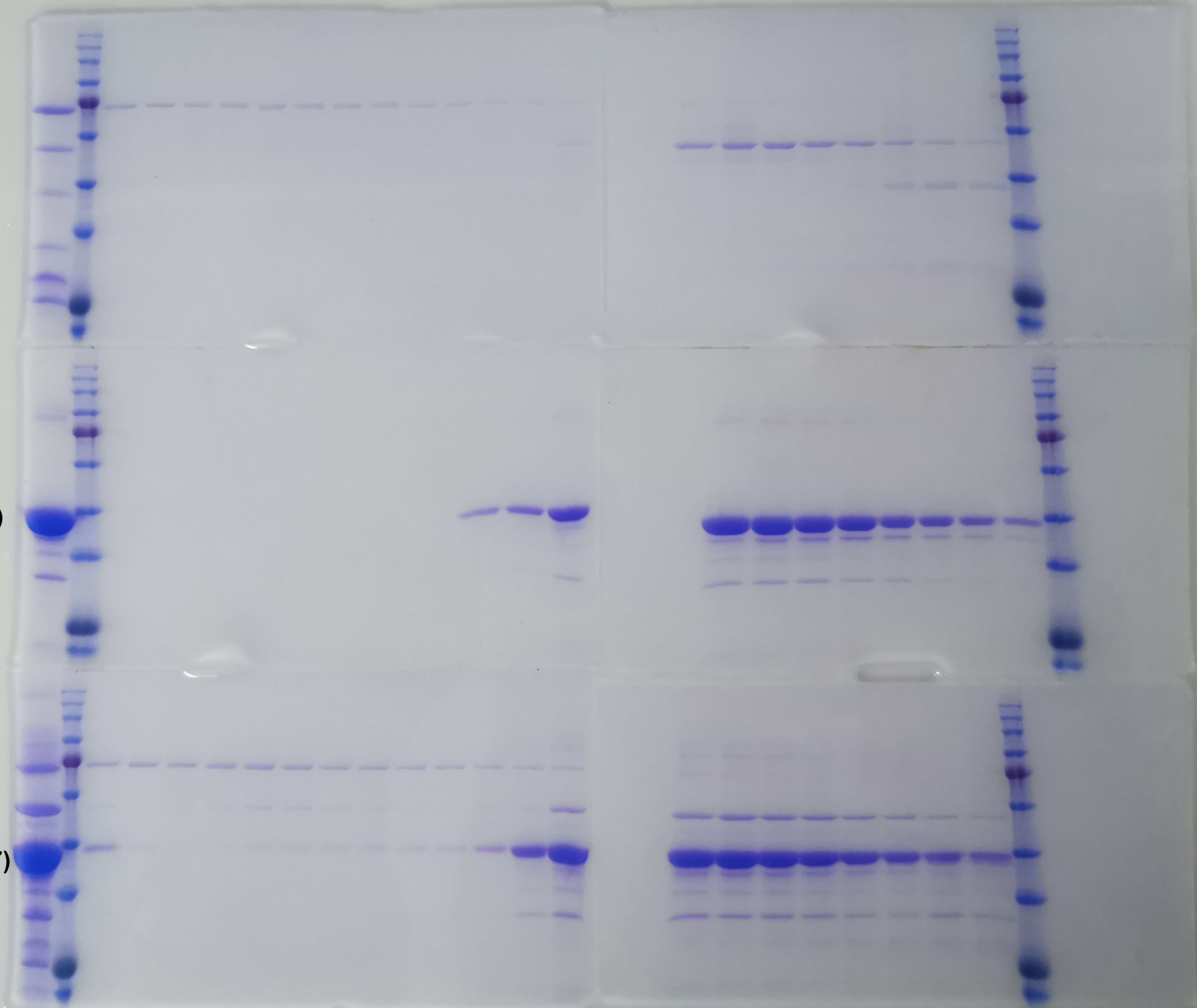

Supplement: Figure 1—figure supplement 1—source data 1. [file elife-86258-fig1-figsupp1-data1.pdf]

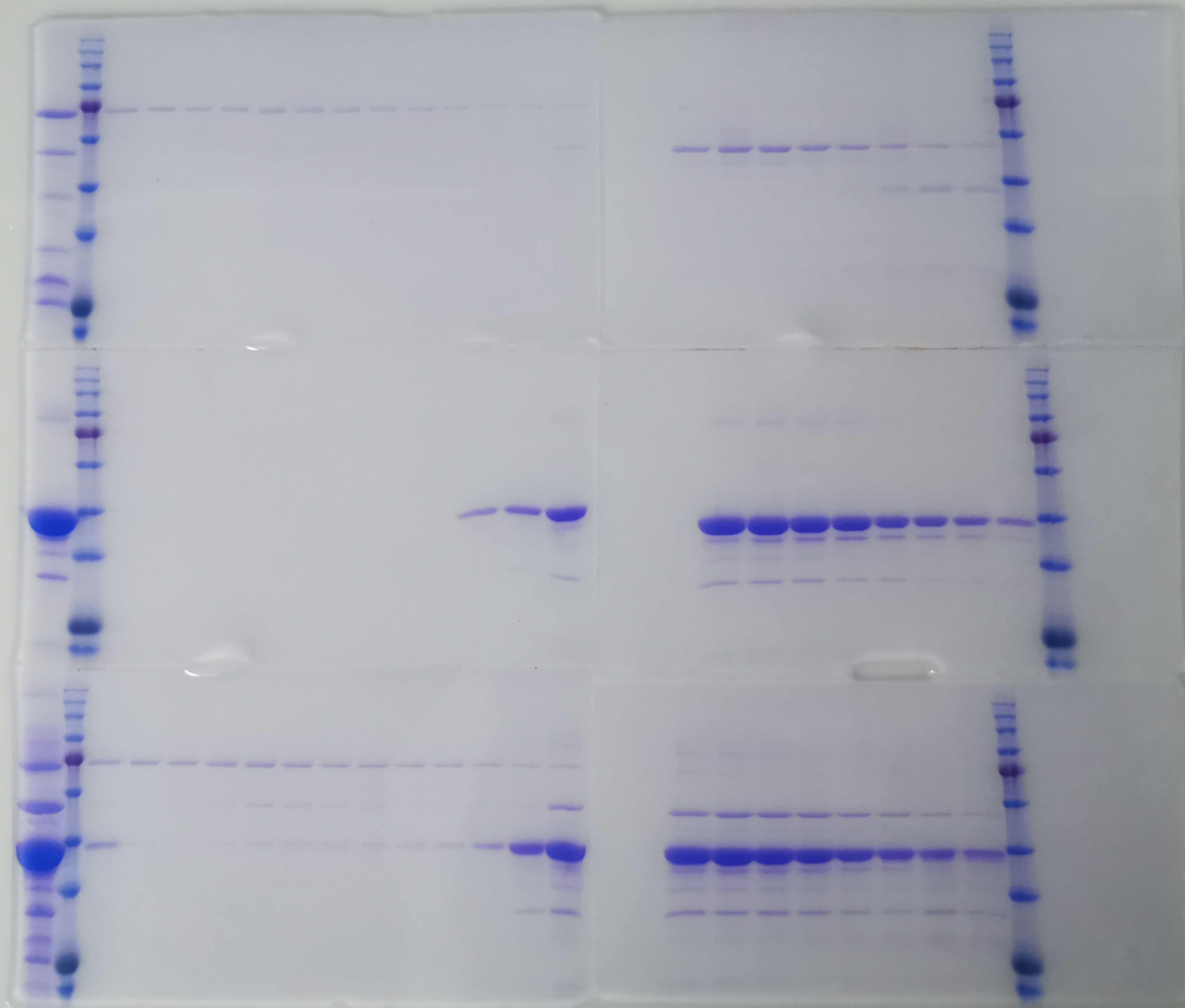

Supplement: Figure 1—figure supplement 1—source data 2. [file elife-86258-fig1-figsupp1-data2.pdf]

**Bcl3  
(1-446)**

**p52  
(1-398)**

**Bcl3  
(1-446)  
+  
p52  
(1-398)**

**p52:p52:Bcl3 Complex  
(1-398)**

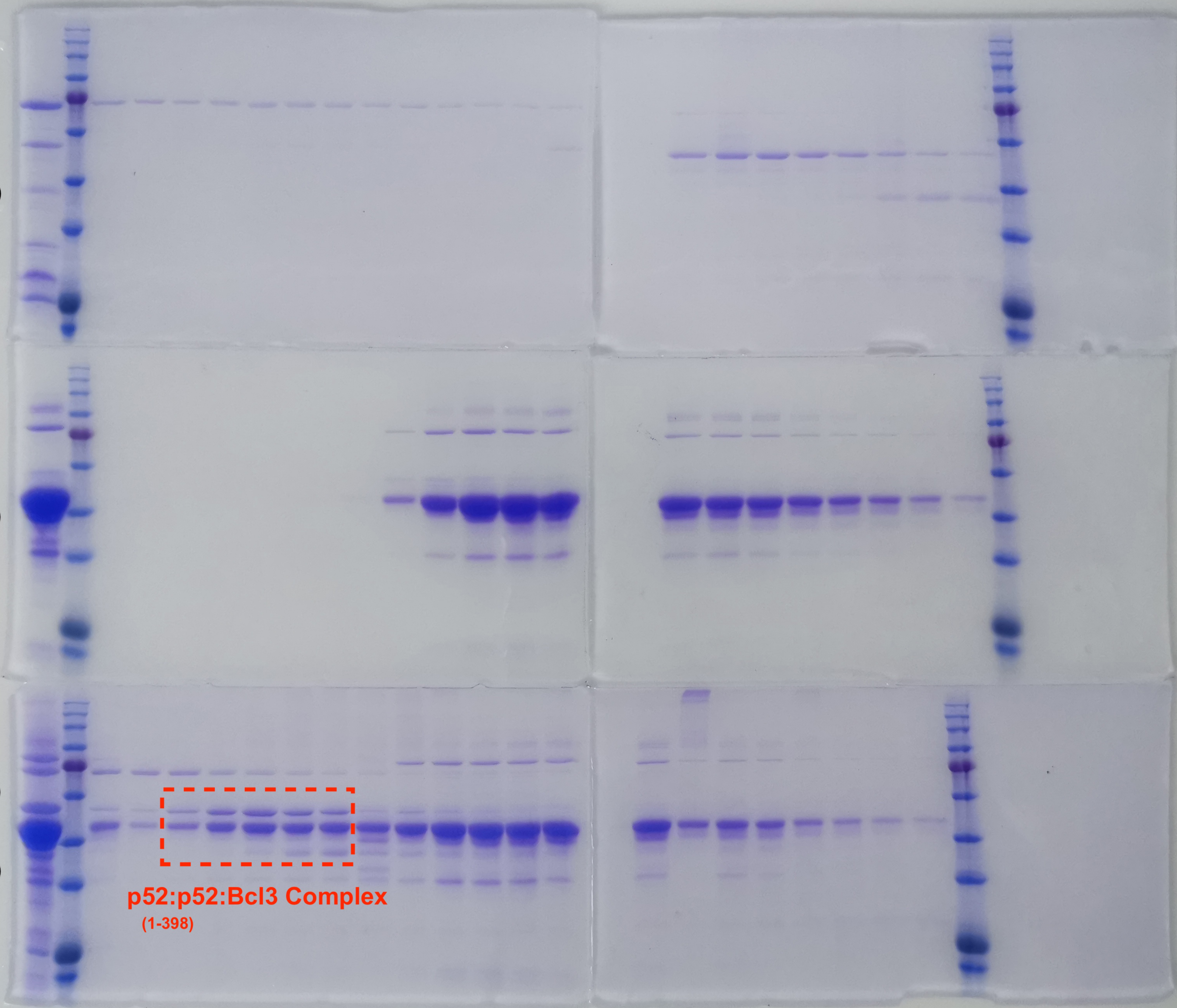

Supplement: Figure 1—figure supplement 1—source data 3. [file elife-86258-fig1-figsupp1-data3.pdf]

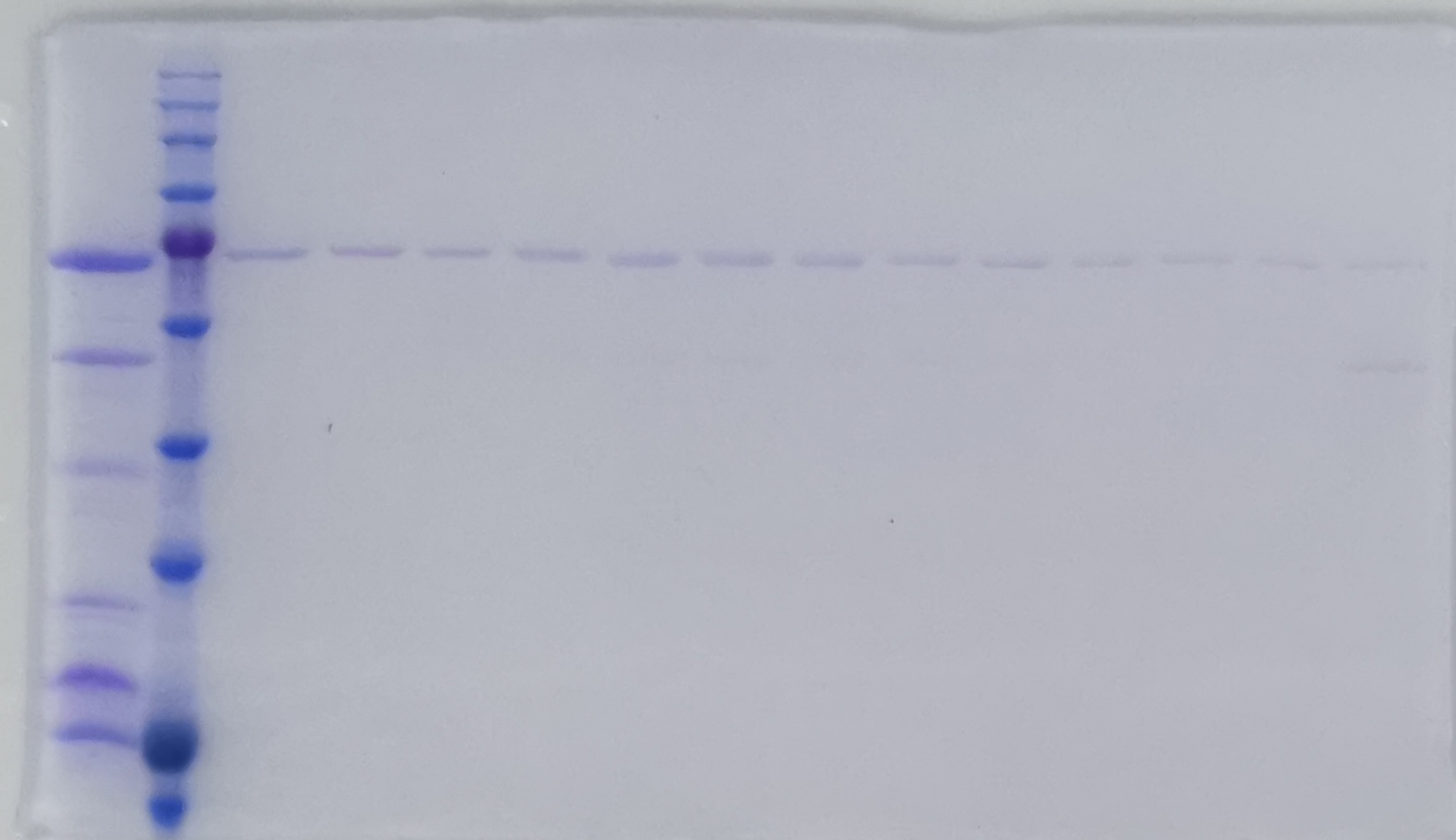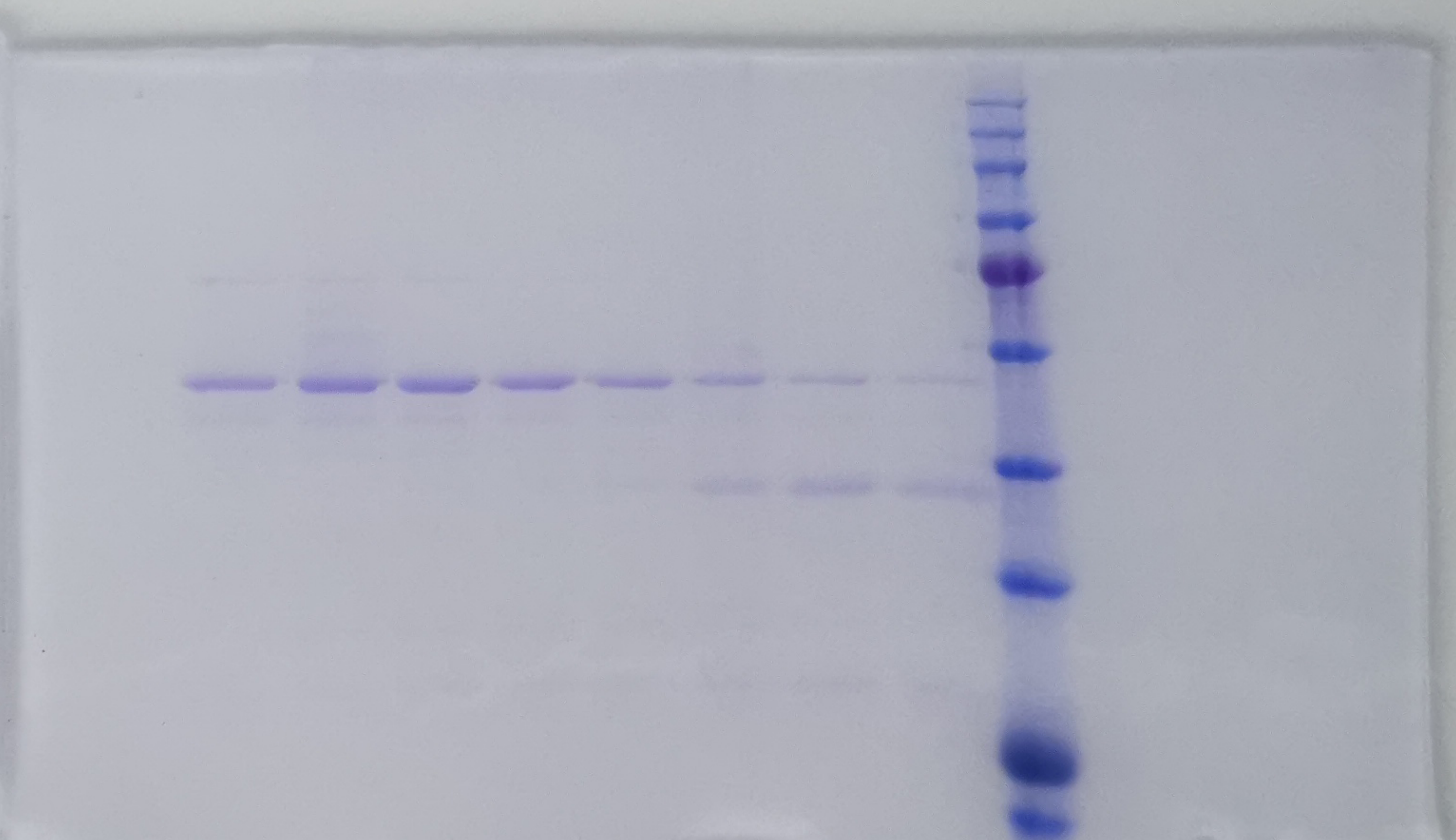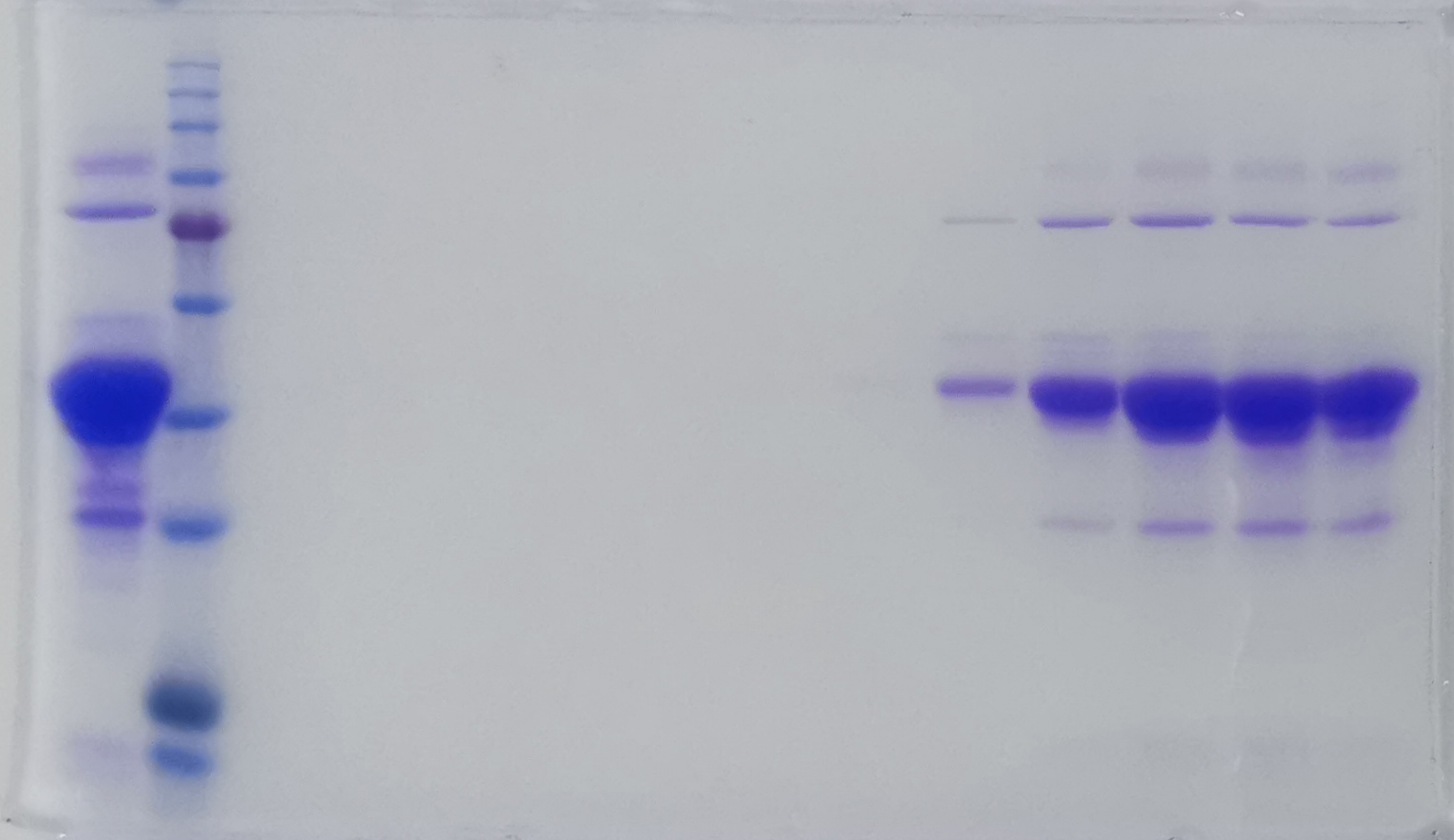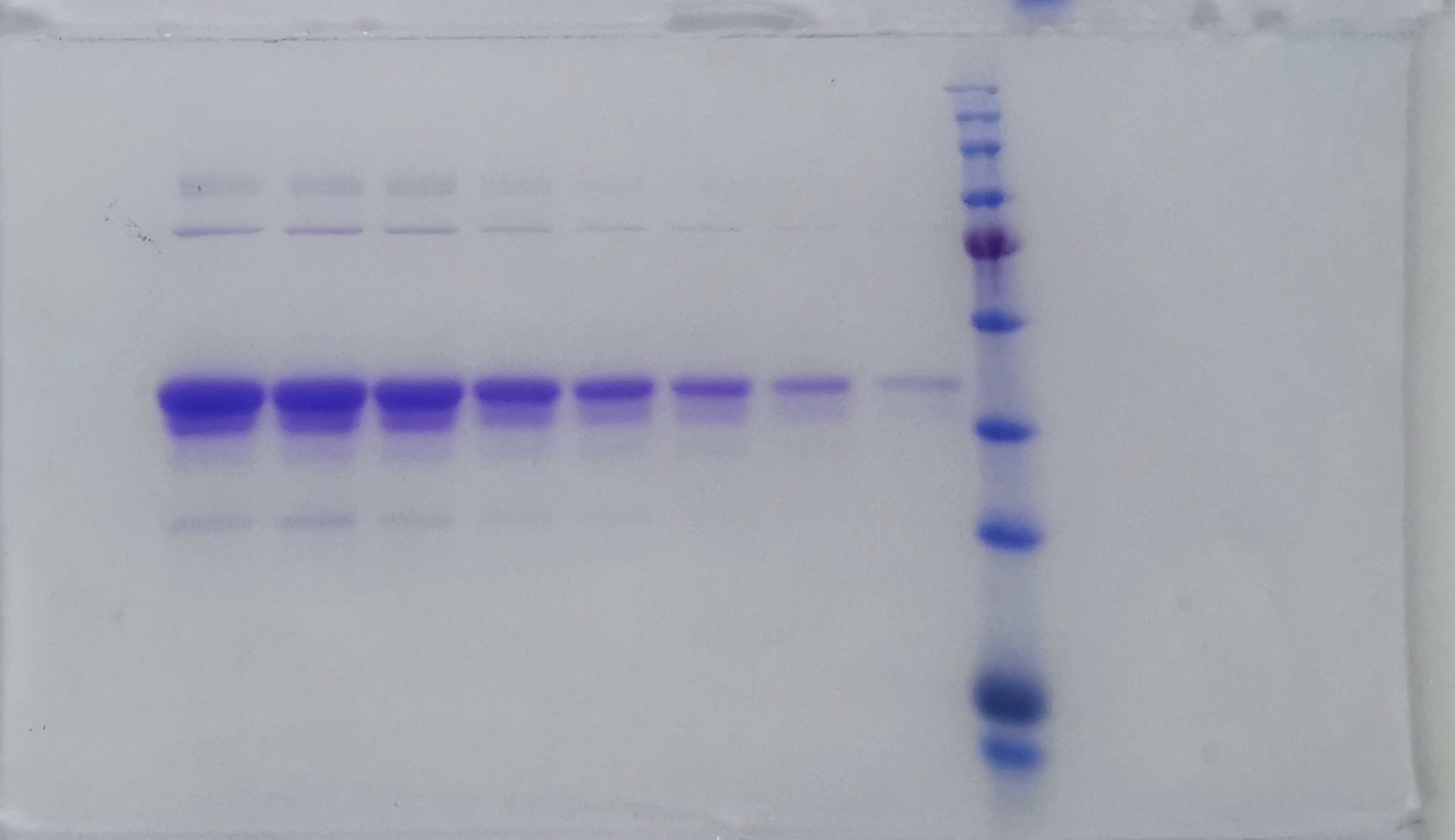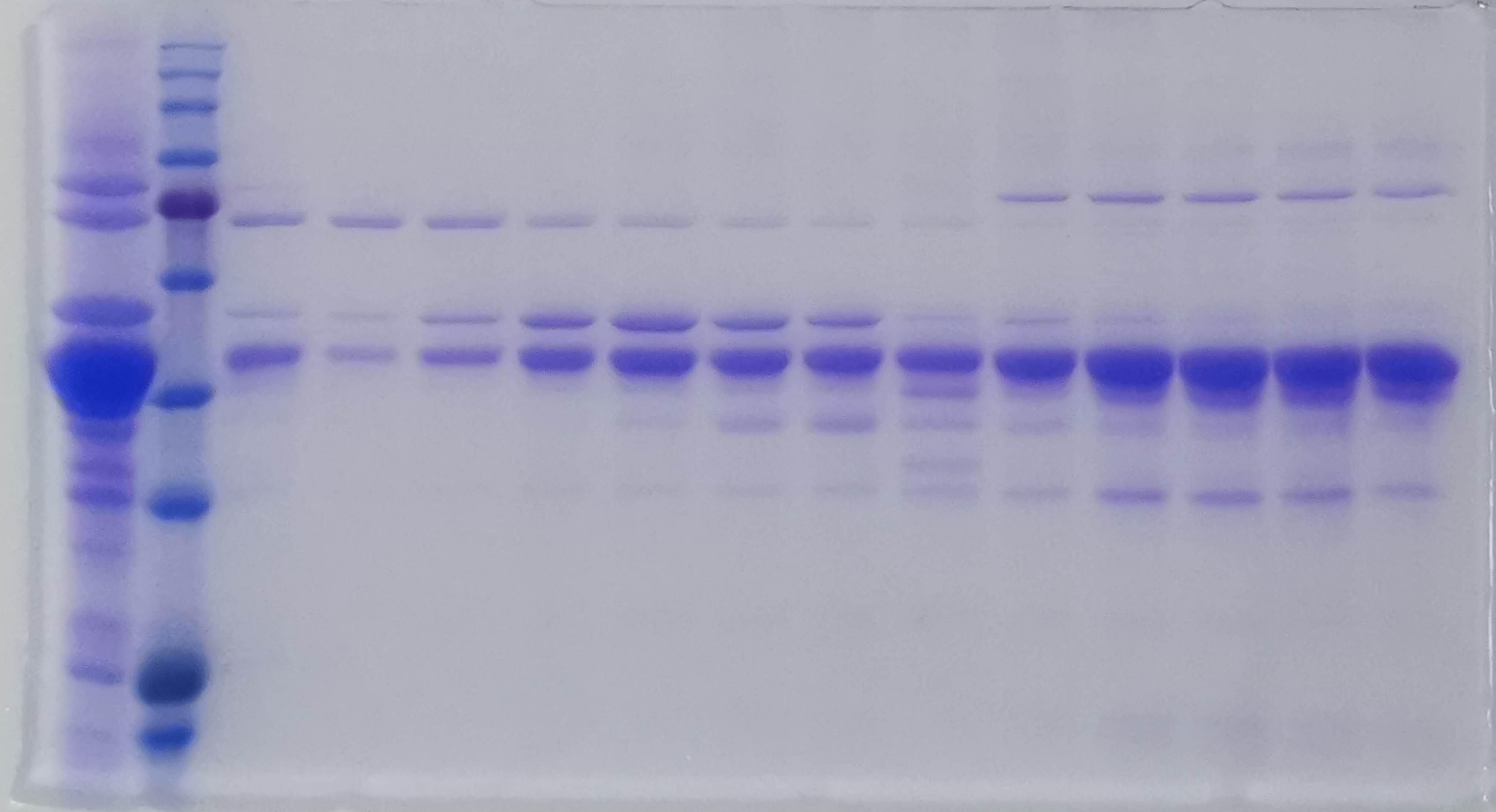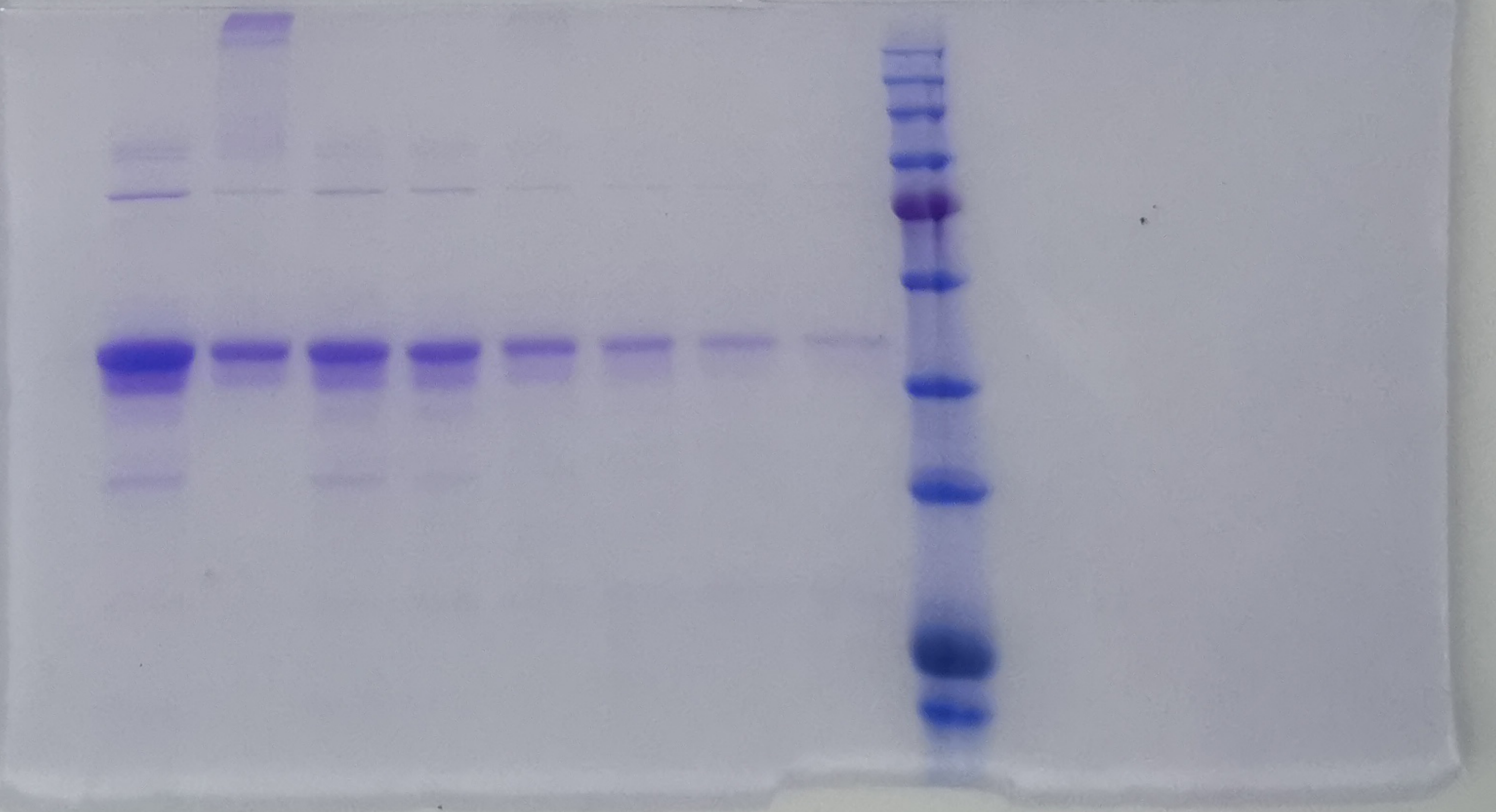

Supplement: Figure 1—figure supplement 1—source data 4. [file elife-86258-fig1-figsupp1-data4.pdf]

Figure 6-figure supplement 2A

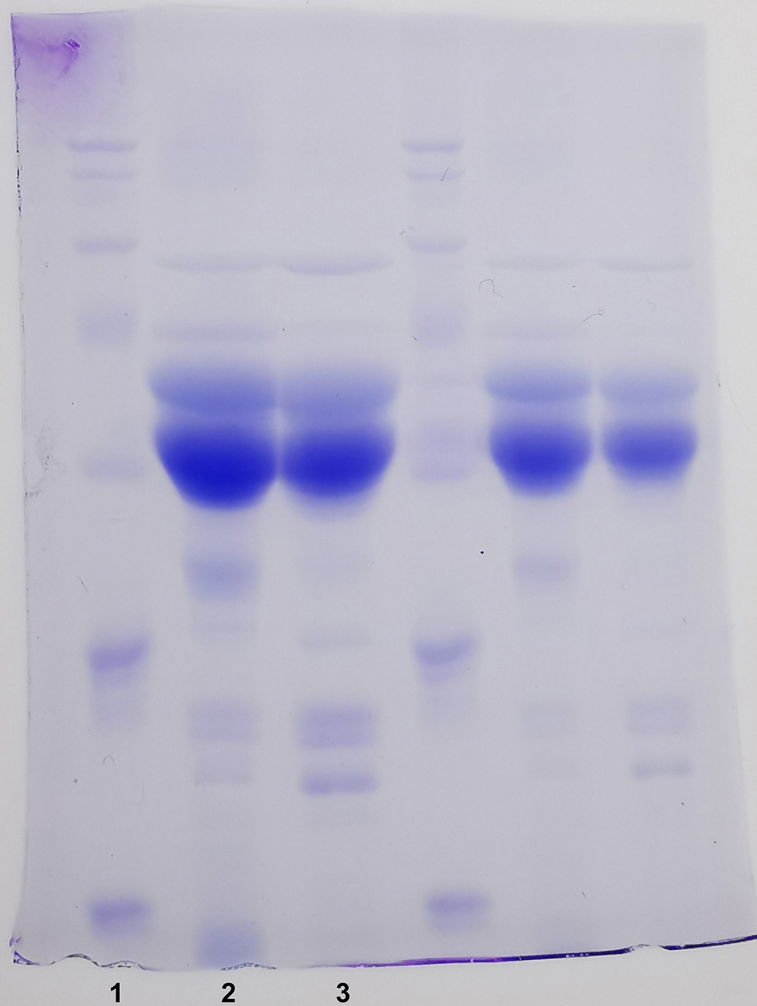

1: MW Std  
2: p52:Bcl3 (WT)  
3: p52:Bcl3 (phospho-mimetic)

Supplement: Figure 6—figure supplement 2—source data 1. [file elife-86258-fig6-figsupp2-data1.pdf]

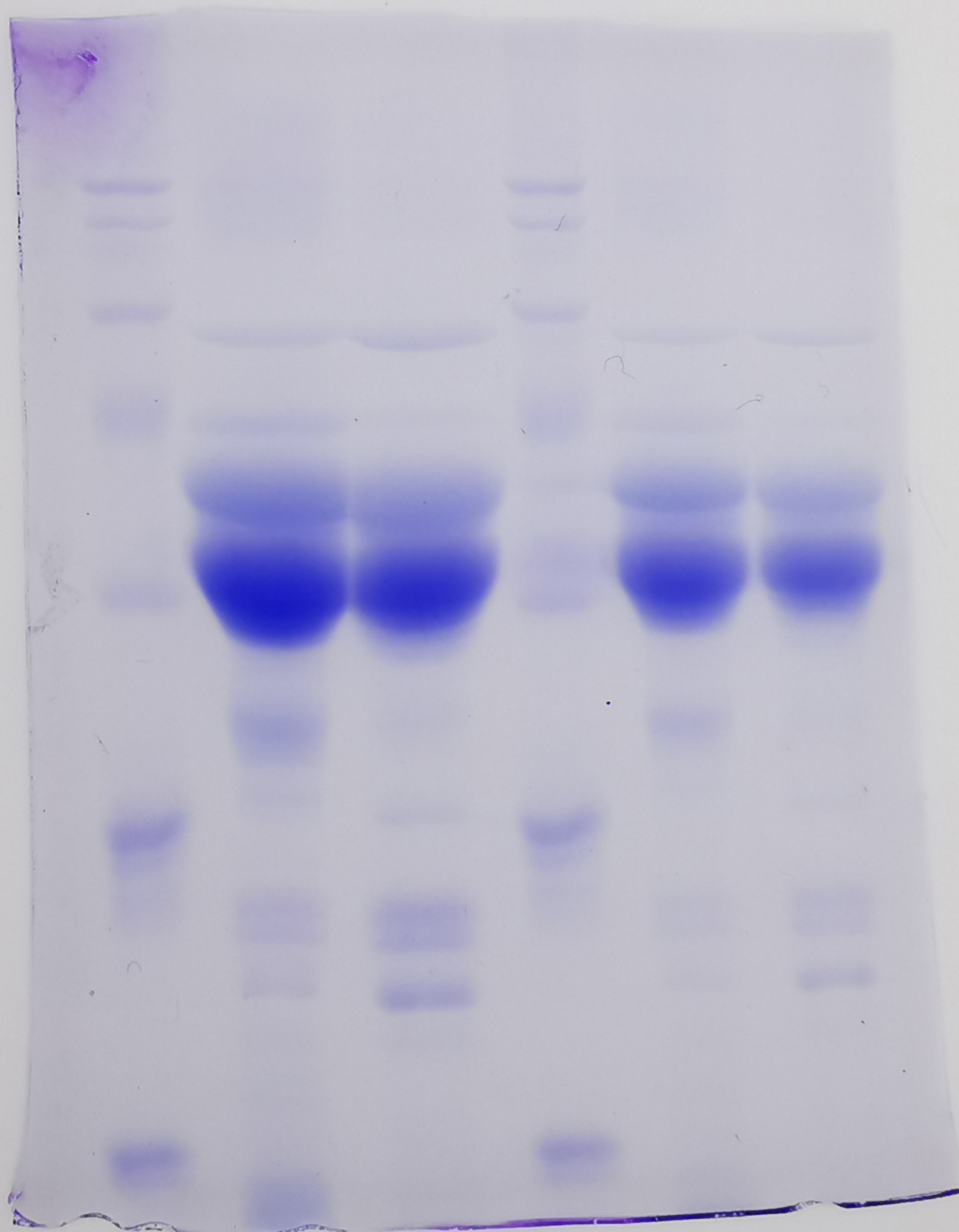

Supplement: Figure 6—figure supplement 2—source data 2. [file elife-86258-fig6-figsupp2-data2.pdf]

**Figure 8-figure supplement 1A**

**1: MW Std**  
**2: p52(WT)**  
**3: p52(K144A)**

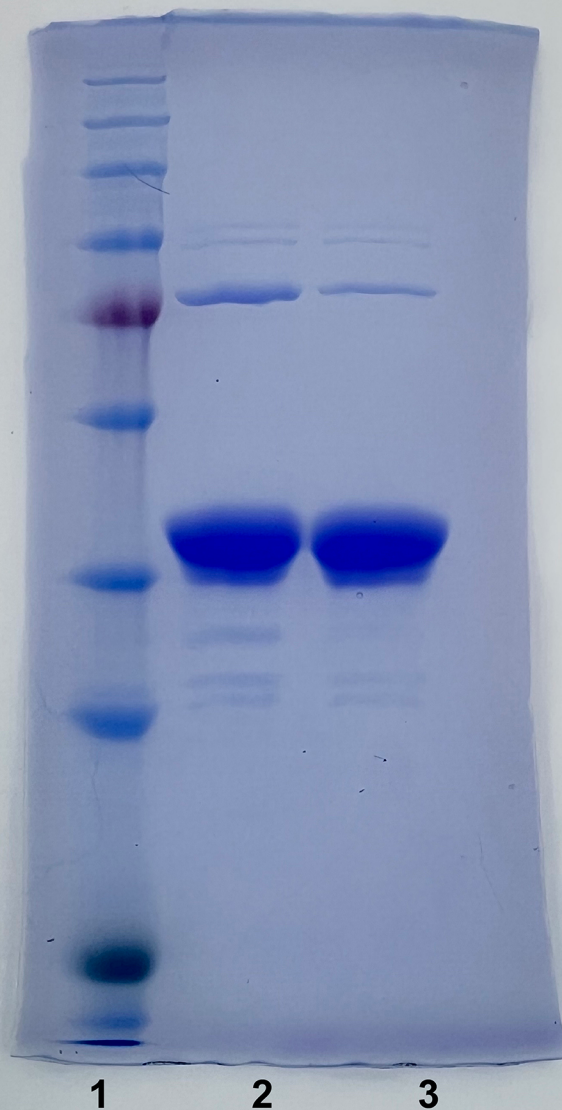

Supplement: Figure 8—figure supplement 1—source data 1. [file elife-86258-fig8-figsupp1-data1.pdf]

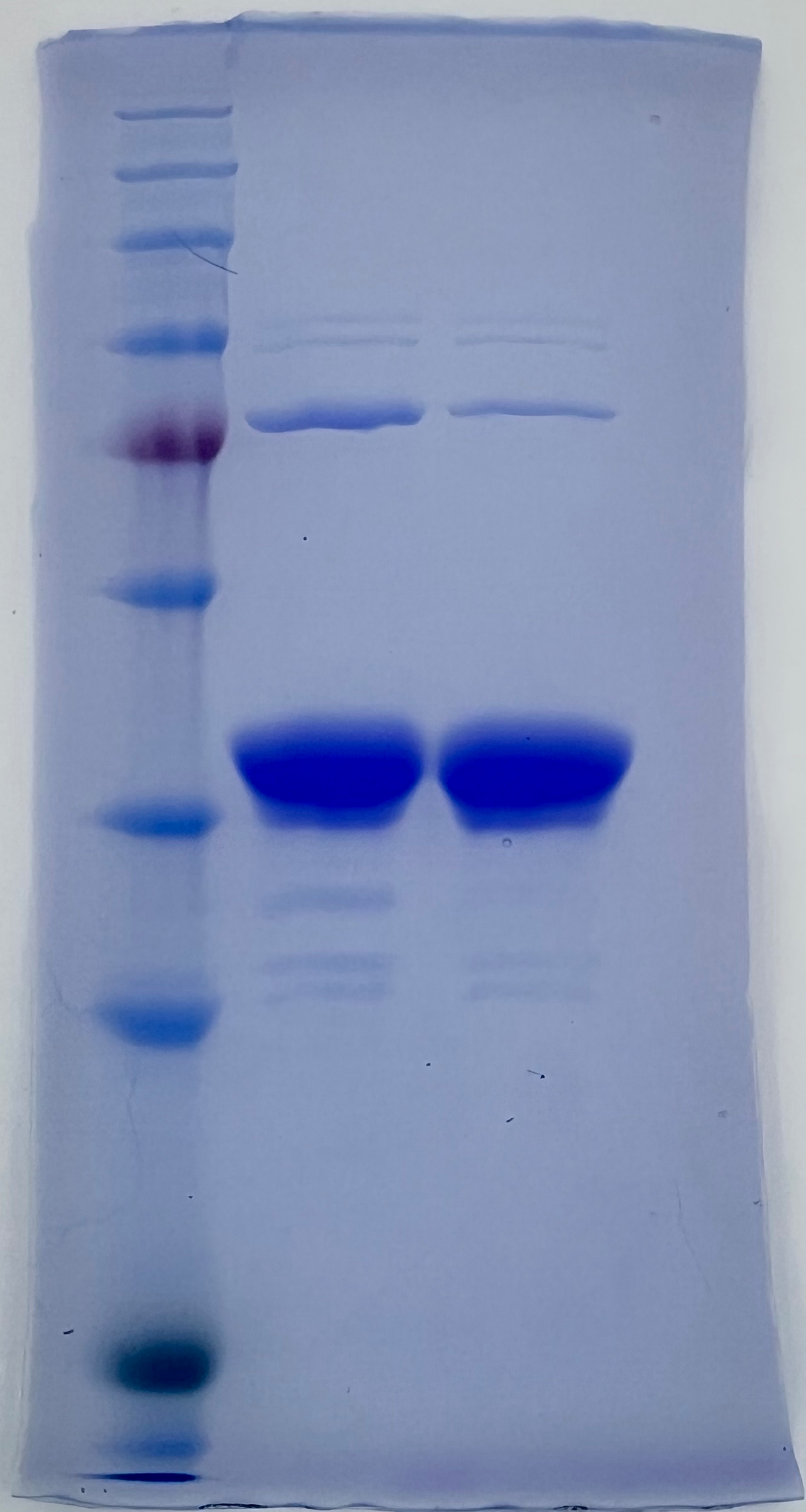

Supplement: Figure 8—figure supplement 1—source data 2. [file elife-86258-fig8-figsupp1-data2.pdf]

**Figure 8-figure supplement 1D**

**1: MW Std**  
**2: p52(WT):Bcl3**  
**3: p52(K144A):Bcl3**

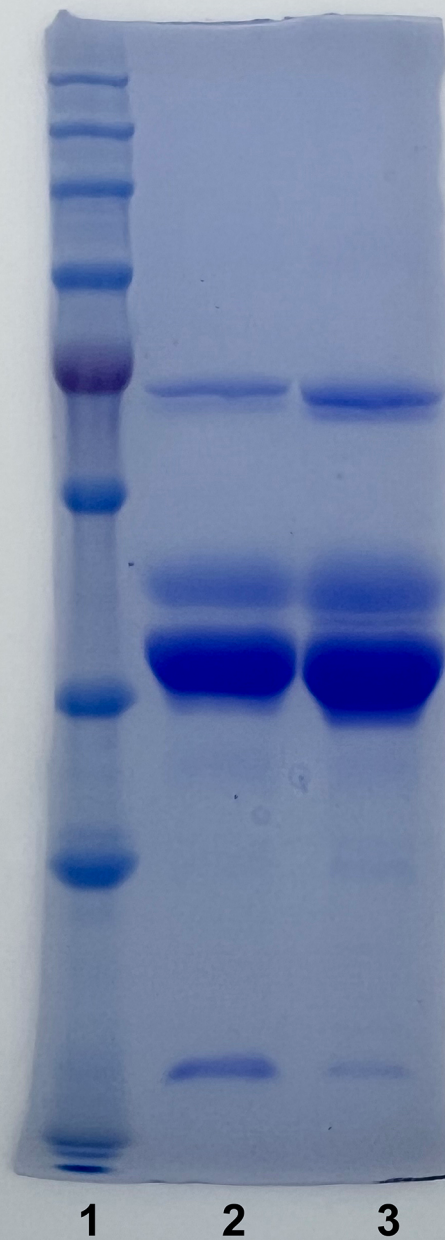

Supplement: Figure 8—figure supplement 1—source data 3. [file elife-86258-fig8-figsupp1-data3.pdf]

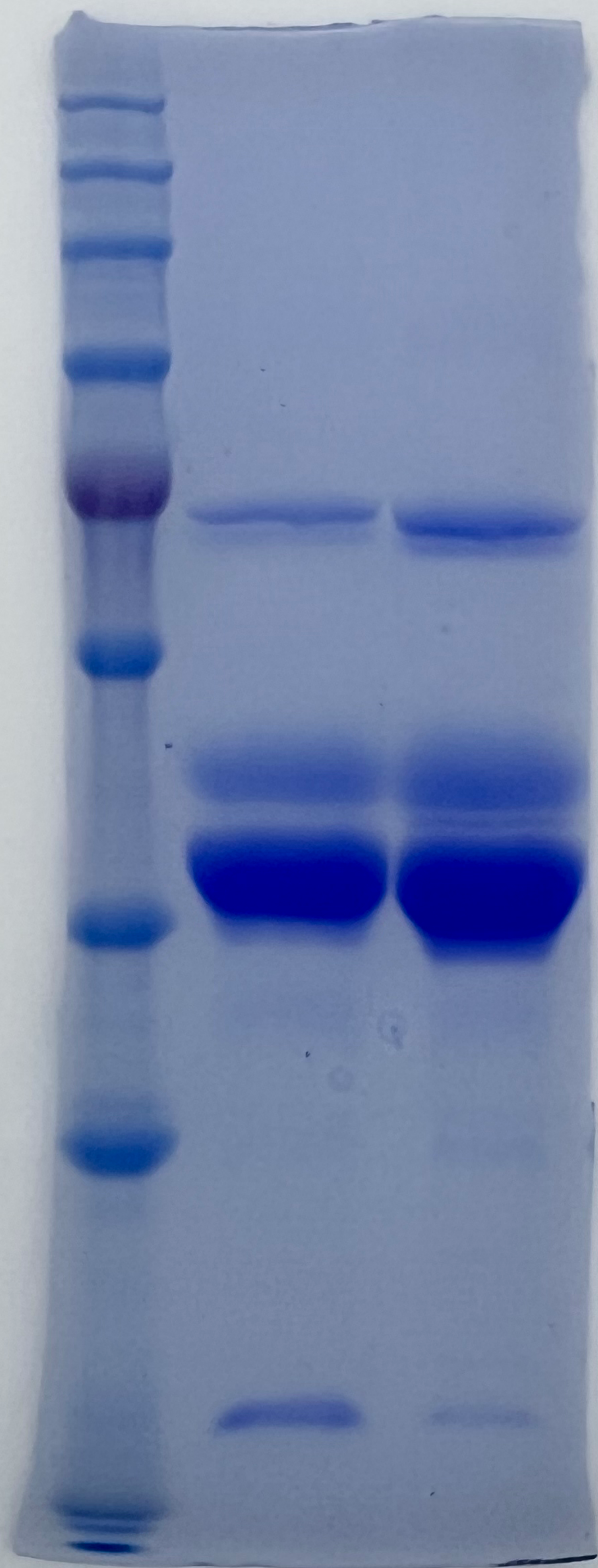

Supplement: Figure 8—figure supplement 1—source data 4. [file elife-86258-fig8-figsupp1-data4.pdf]
