# Supplementary material for: Structures of NF-κB p52 homodimer-DNA complexes rationalize binding mechanisms and transcription activation
Source: eLife. 2023 Feb 13;12:e86258. doi: 10.7554/eLife.86258 (PMC9991059; doi:10.7554/eLife.86258)
Supplement: Supplementary file 1. [file elife-86258-supp1.docx]

**Supplementary File 1.** **Nucleotide sequences of** **PSel-κB DNA variants used in crystallization.**

|  | **PSel (natural G/C-centric)** | **PSel (mutant A/T-centric)** | **PSel (-1/+1 swap)** |
| --- | --- | --- | --- |
| 12 bp | 5’-G**GGGGTGACCCC**-3’  3’-C**CCCCACTGGGG**-5’ | 5’-G**GGGGTAACCCC**-3’  3’-C**CCCCATTGGGG**-5’ | 5’-G**GGGGAGTCCCC**-3’  3’-C**CCCCTCAGGGG**-5’ |
| 13 bp  (5’ extra flanking) | 5’-AG**GGGGTGACCCC**-3’  3’-TC**CCCCACTGGGG**-5’ | 5’-AG**GGGGTAACCCC**-3’  3’-TC**CCCCATTGGGG**-5’ | 5’-AG**GGGGAGTCCCC**-3’  3’-TC**CCCCTCAGGGG**-5’ |
| 13 bp | 5’-G**GGGGTGACCCC**T-3’  3’-C**CCCCACTGGGG**A-5’ | 5’-G**GGGGTAACCCC**T-3’  3’-C**CCCCATTGGGG**A-5’ | 5’-G**GGGGAGTCCCC**T-3’  3’-C**CCCCTCAGGGG**A-5’ |
| 14 bp | 5’-AG**GGGGTGACCCC**T-3’  3’-TC**CCCCACTGGGG**A-5’ | 5’-AG**GGGGTAACCCC**T-3’  3’-TC**CCCCATTGGGG**A-5’ | 5’-AG**GGGGAGTCCCC**T-3’  3’-TC**CCCCTCAGGGG**A-5’ |
| 15 bp  (5’ extra flanking) | 5’-AAG**GGGGTGACCCC**T-3’  3’-TTC**CCCCACTGGGG**A-5’ | 5’-AAG**GGGGTAACCCC**T-3’  3’-TTC**CCCCATTGGGG**A-5’ | 5’-AAG**GGGGAGTCCCC**T-3’  3’-TTC**CCCCTCAGGGG**A-5’ |
| 15 bp | 5’-AG**GGGGTGACCCC**TT-3’  3’-TC**CCCCACTGGGG**AA-5’ | 5’-AG**GGGGTAACCCC**TT-3’  3’-TC**CCCCATTGGGG**AA-5’ | 5’-AG**GGGGAGTCCCC**TT-3’  3’-TC**CCCCTCAGGGG**AA-5’ |
| 16 bp | 5’-AAG**GGGGTGACCCC**TT-3’  3’-TTC**CCCCACTGGGG**AA-5’ | 5’-AAG**GGGGTAACCCC**TT-3’  3’-TTC**CCCCATTGGGG**AA-5’ | 5’-AAG**GGGGAGTCCCC**TT-3’  3’-TTC**CCCCTCAGGGG**AA-5’ |
| 18 bp | 5’-GAAG**GGGGTGACCCC**TTG-3’  3’-CTTC**CCCCACTGGGG**AAC-5’ | 5’-GAAG**GGGGTAACCCC**TTG-3’  3’-CTTC**CCCCATTGGGG**AAC-5’ | 5’-GAAG**GGGGAGTCCCC**TTG-3’  3’-CTTC**CCCCTCAGGGG**AAC-5’ |
| 20 bp | 5’-GGAAG**GGGGTGACCCC**TTGG-3’  3’-CCTTC**CCCCACTGGGG**AACC-5’ | 5’-GGAAG**GGGGTAACCCC**TTGG-3’  3’-CCTTC**CCCCATTGGGG**AACC-5’ | 5’-GGAAG**GGGGAGTCCCC**TTGG-3’  3’-CCTTC**CCCCTCAGGGG**AACC-5’ |

κB sites are underlined with the central position 0 colored in red and the swap of −1 and +1 positions colored in green.
