# Supplementary material for: Structures of NF-κB p52 homodimer-DNA complexes rationalize binding mechanisms and transcription activation
Source: eLife. 2023 Feb 13;12:e86258. doi: 10.7554/eLife.86258 (PMC9991059; doi:10.7554/eLife.86258)
Supplement: Supplementary file 2. [file elife-86258-supp2.docx]

**Supplementary File 2.** **Assembly analysis of p52-DNA complexes made by PDBePISA.**

| **Structure** | **p52-PSel**  **(natural G/C-centric)**  **(7CLI)** | **p52-PSel**  **(mutant A/T-centric)**  **(7VUQ)** | **p52-PSel**  **(−1/+1 swap)**  **(7VUP)** | **p52-PSel**  **(mutant 13-mer A/T-centric)**  **(7W7L)** |
| --- | --- | --- | --- | --- |
| p52 construct | aa 1-398 | aa 1-398 | aa 1-398 | aa 1-327 |
| DNA length | 18 bp | 18 bp | 18 bp | 13 bp |
| Surface area, Å^2^ | 36480 | 36190 | 35710 | 34710 |
| Buried area in p52 dimer interface, Å^2^ | 1391 | 1364 | 1336 | 1353 |
| Buried area in protein-DNA interface, Å^2^ | 3070 | 3210 | 3417 | 3270 |
| Total number of protein-DNA H-bond | 22 | 23 | 23 | 30 |
